# Influenza virus infections among patients attending emergency department according to main reason to presenting to ED: A 3-year prospective observational study during seasonal epidemic periods

**DOI:** 10.1371/journal.pone.0182191

**Published:** 2017-08-16

**Authors:** Enrique Casalino, Stephanie Antoniol, Nadhira Fidouh, Christophe Choquet, Jean-Christophe Lucet, Xavier Duval, Benoit Visseaux, Laurent Pereira

**Affiliations:** 1 Assistance Publique-Hôpitaux de Paris (AP-HP), Groupe Universitaire Paris Nord Val de Seine, Emergency Department, Paris, France; 2 Université Paris Diderot, Sorbonne Paris Cité, EA 7334 Recherche clinique coordonnée ville-hôpital, Méthodologies et Société (REMES), Paris, France; 3 Study Group for Efficiency and Quality of Emergency Departments and Non-Scheduled Activities Departments, Paris, France; 4 Assistance Publique-Hôpitaux de Paris (AP-HP), Groupe Universitaire Paris Nord Val de Seine, Virology Department, Paris, France; 5 Assistance Publique-Hôpitaux de Paris (AP-HP), Bichat-Claude Bernard Hospital, Infection Control Unit, Paris, France; 6 Inserm CIC-1425, AP-HP, Hôpital Universitaire Bichat, Paris, France; 7 IAME, UMR 1137, INSERM, Université Paris Diderot, Sorbonne Paris Cité, Paris, France; Kliniken der Stadt Köln gGmbH, GERMANY

## Abstract

**Objective:**

The role of influenza virus in patients presenting at ED during seasonal-epidemic periods has not previously been specified. Our objective was to determine its frequency according to clinical presentation.

**Methods:**

This is a prospective observational study conducted during three-consecutive seasonal Influenza epidemics (2013–2015), including patients presenting i) community-acquired pneumonia (CAP); ii) severe acute symptoms (SAS): respiratory failure (RF), hemodynamic failure (HF), cardiac failure (CF), and miscellaneous symptoms (M); iii) symptoms suggesting influenza (PSSI). Patients were tested for influenza using specific PCR on naso-pharyngeal swabs.

**Results:**

Of 1,239 patients, virological samples were taken from 784 (63.3%), 213 (27.2%) of whom were positive for the influenza virus: CAP 52/177 (29.4%), SAS 115/447 (25.7%) and PSSI 46/160 (28.8%) (p = 0.6). In the SAS group positivity rates were: RF 76/263 (28.9%), HF 5/29 (17.2%), CF 15/68 (22.1%), and M 19/87 (21.8%) (p = 0.3). Among the major diagnostic categories, the influenza virus positivity rates were: asthma 60/231 (26%), acute exacerbation of chronic obstructive pulmonary disease 18/86 (20.9%), HIV 5/21 (23.8%) and cardiac failure 33/131 (25.2%). The positivity of the samples has not been associated (p>0.1) nor the presence of signs of severity or admission rate in medical ward nor intensive care unit.

**Conclusions:**

Our results indicate that during seasonal influenza epidemics, Influenza virus-positivity rate is similar in patients attending ED for influenza-compatible clinical features, patients with acute symptoms including pneumonia, respiratory, hemodynamic and cardiac distress, and patients presenting for acute decompensation of chronic respiratory and cardiac diseases.

## Introduction

Seasonal influenza occurs in epidemic peaks during the winter periods. Particular climate conditions, including cold and humidity, school holidays, and the characteristics of the likely circulating viral strain and vaccination coverage, are associated with epidemic peaks that vary in intensity in terms of the number of patients and the severity of the observed cases [1].

There is an increase in the number of acute asthma episodes during the winter period [2], as well as in acute exacerbations of chronic obstructive pulmonary disease (AE-COPD) [3], decompensation of cardiac diseases [4], hospitalizations, particularly among the elderly [5], and in pulmonary, cardiovascular, and neuromuscular complications [6–8]. This means that the reception capacities of the emergency services and intensive care units (ICU) can rapidly become increasingly stretched [9].

Determination of the number of lengthy hospital stays, primarily for respiratory and hemodynamic decompensations related to respiratory viruses, and to influenza viruses in particular, is a strategic priority, in order to promote improvements in prevention, diagnostics, and therapeutics, as well as in care delivery [10]. The influenza virus has been identified as the causative agent of 2.2–18% of lung diseases [11–13], up to 10% of asthma [2] and AE-COPD episodes [14], 4–33% of patient admissions to ICU for community-acquired pneumonia (CAP) [15], and 3.4% of all admissions to ICU [16]. Nevertheless, to our knowledge, no study has evaluated the role of the influenza virus in episodes of respiratory, cardiac, or hemodynamic distress during periods of influenza epidemic in the ED.

The objective of this study was to identify the number of patients infected with the influenza virus among those presenting at the ED with pneumonia or severe acute symptoms or presenting symptoms suggesting Influenza, as well as the impact of the influenza virus on the severity of illness and the fate of the patients.

## Material and methods

### Study design and setting

This was a prospective observational study conducted as part of a continuous quality improvement program for the diagnosis and treatment of viral infections in the ED of the hospital Bichat—Claude Bernard (BCB), Paris, France. The BCB is an academic, 1,000-bed hospital, and its ED receives 80,000 visits each year.

### Selection of participants

Adults attending the BCB ED during three consecutive periods of seasonal influenza epidemic (2013–2015) were included. The National Institute for Public Health Surveillance annually determines the beginning and the end of the seasonal influenza epidemic, according to the number of cases reported by the influenza surveillance networks, [17].

In accordance with the inclusion criteria, three groups were defined on the basis of the initial reason for presentation in the ED. Groups were defined by ED physician during ED stay: (1) Patients presenting with acute respiratory symptoms suggesting CAP (CAP group); (2) Patients presenting with severe acute symptoms (SAS group) [18]: polypnea, cyanosis, oxygen saturation Spa02 <95%, pneumonia, wheeze, tachycardia, hypotension, areas of mottled skin, malaise, change in mental status, and oliguria; and (3) patients with symptoms suggesting Influenza virus infection (fever and cough or myalgias or rhinorrhea) and with underlying conditions for severe influenza (PSSI group): age ≥65 years, asthma, bronchopulmonary dysplasia, cystic fibrosis, chronic respiratory failure, cardiac failure, cardiac valvulopathy, congenital heart disease, cardiovascular disease, renal failure, nephrotic syndrome, sickle-cell anemia, hepatic failure, diabetes, systemic corticosteroid therapy, leukemia or lymphoma, immunosuppression, cancer, HIV infection, CD4 lymphocytes count, obesity, and pregnancy [19]. Patients in the SAS group were classified into four subgroups on the basis of their main reason for visiting the ED: a) respiratory failure (RF); b) hemodynamic failure (HF); c) cardiac failure (CF); and d) miscellaneous (M).

For each patient meeting the inclusion criteria, a standard case report form, which included demographic and clinical data, was completed by emergency physicians as a part of a quality of care program. The data were prospectively recorded and collected from the computerized emergency database system (Urqual^®^, McKesson International, Paris, France), and the final disposition decision was recorded (Medical or Intensive Care Unit).

The rate of influenza in presentations of asthma, acute exacerbation of chronic obstructive pulmonary disease (AE-COPD), HIV infected patients and acute decompensation of cardiac failure was calculated on the basis of the final diagnosis of emergency (ICD-10).

### Interventions

Nasopharyngeal samples were obtained from patients with CAP, severe acute conditions, or underlying clinical conditions for severe influenza at the time of study enrollment. It was also possible to include some patients with an indication of hospitalization, patients residing in elderly care centers, healthcare workers, and people living with a person with underlying clinical conditions for severe influenza.

Swab samples were placed in universal vials in universal transport media (Sigma-Virocult^®^, MW951S) and stored at + 4°C if not tested immediately. All samples were processed for influenza detection within 36 hours, using the Xpert^®^ Flu PCR test (Cepheid, California, USA.). This test also allowed the classification of influenza viruses.

Bacteriological findings were obtained from electronic clinical records.

### Ethics statement

Data collection and storage by the Urqual^®^ Emergency Database was approved by the French National Commission for Data Protection and Liberties. Anonymized data was extracted from the CNR-M database. The Emergency Ethics Committee for Biomedical Research of Assistance Publique-Hôpitaux de Paris approved this study.

### Analysis

In order to describe the study population, quantitative variables have been expressed as mean and standard deviation, and qualitative variables as numbers of patients and percentages. A Chi 2 or Fisher’s test and a Student’s t-test or Wilcoxon test were used to compare qualitative and quantitative variables between study groups. The significance threshold was set at p = 0.05. Statistical analyses were performed using Statistica^®^ software (StatSoft).

## Results

### Characteristics of study subjects

**Fig 1:**
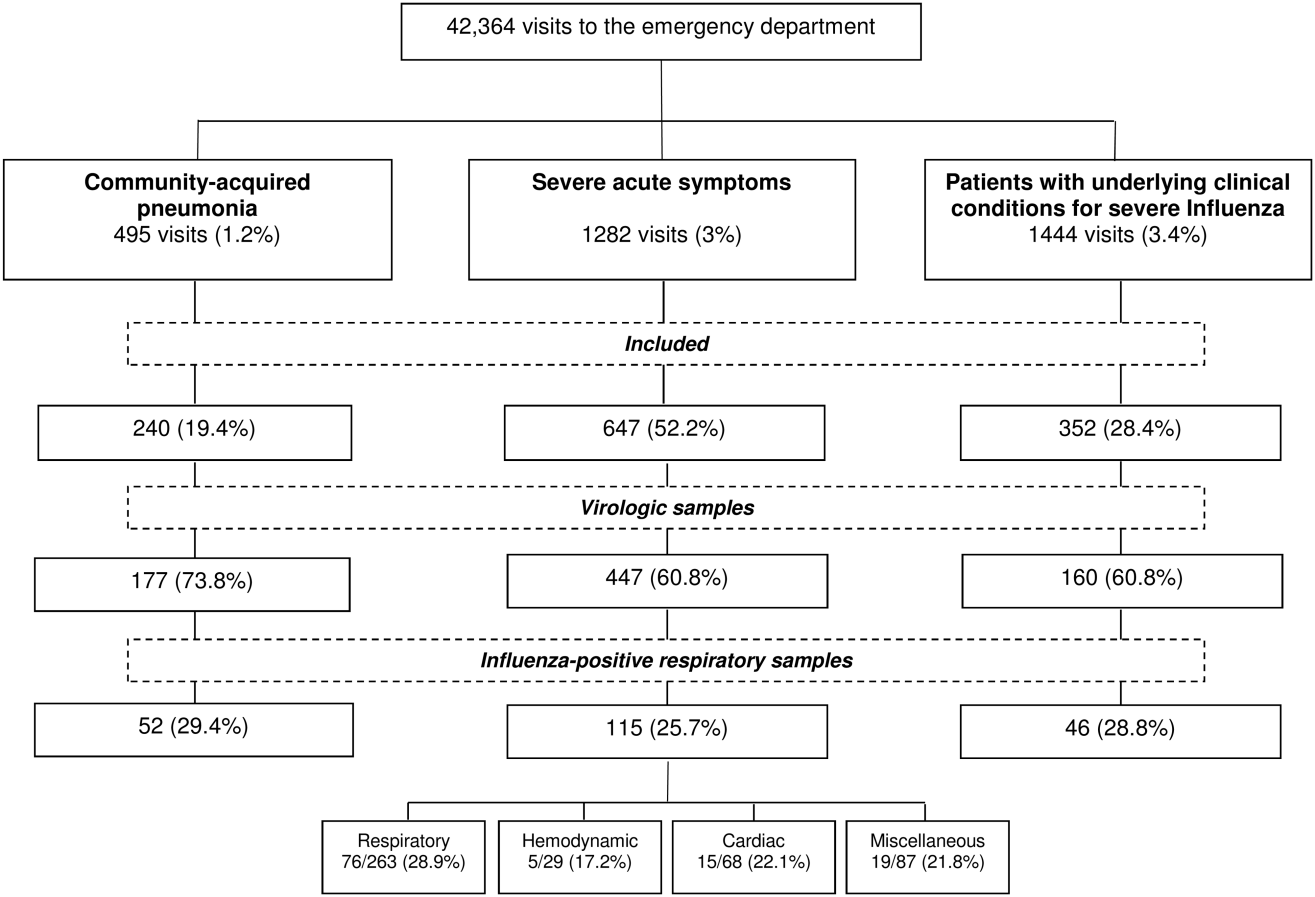
Study flowchart.

During the three consecutive periods, 42,364 ED visits were recorded. On the basis of the reason for presentation and the ICD-10 coding, 3,221 visits (7.6%) were considered for inclusion in our study. Of these, 1,239 (38.5%) were actually included, as follows: 240 (19.4%) cases of CAP, 647 (52.2%) patients with SAS, and 352 (28.4%) cases of PSSI. Fig 1 shows a flowchart relating to the study population.

Table 1 shows the main characteristics of the patients included, according to the reason for ED presentation. It can be observed that the CAP and SAS groups more frequently had underlying clinical conditions, putting them at risk of severe influenza, than did the group of patients with clinical symptoms suggestive of influenza, but without signs of seriousness.

**Table 1:** The main characteristics of the study groups.

	Community-acquired pneumonia		Severe acute symptoms		Patients with underlying conditions for severe influenza		P
	n = 240		n = 647		n = 352		
	moy±DS		moy±DS		moy±DS		
	n	%	n	%	n	%	
**Risk factors for severe influenza infection**							
Age	68.5±18.7		70.6±18.5		55±21.1		0.002
Age ≥65 years	153	63.8	444	68.6	127	36.1	0.00001
Asthma	44	18.7	259	40.6	38	11.2	0.00001
Bronchopulmonary dysplasia	5	2.1	20	3.1	1	0.3	0.01
Cystic fibrosis	23	9.8	34	5.3	13	3.9	0.009
Chronic respiratory failure	19	8.1	94	14.8	10	3	0.000001
Cardiac failure	27	11.5	126	19.8	20	5.9	0.000001
Cardiac valvulopathy	9	3.8	31	4.9	4	1.2	0.01
Congenital heart disease	3	1.3	14	2.2	4	1.2	0.4
Cardiovascular disease	14	6	57	9	10	3	0.002
Renal failure	21	8.9	68	10.7	19	5.6	0.03
Nephrotic syndrome	1	0.4	1	0.2	4	1.2	0.09%
Sickle-cell anemia	0	0	1	0.2	3	0,9	0.1
Hepatic failure	1	0.4	3	0.5	0	0	0.5
Diabetes	37	15.7	123	19.3	43	12.7	0.03
Systemic corticosteroid therapy	14	6	43	6.8	18	5.3	0.7
Leukemia or Lymphoma	6	2.6	5	0.8	2	0.6	0.05
Immunosuppression	14	6	24	3.8	18	5.3	0.3
Cancer	11	4.7	31	4.9	11	3.3	0.5
HIV infection	10	4.3	14	2.2	11	3.3	0.2
CD4 count	196±347		145±174		15–19		0.4
Obesity	11	4.70%	44	6.9	9	2.7	0.02
Pregnancy	1	0.4	6	0,9	4	1.2	0.6
At least one of the above	177	75.3	640	52.7	339	27.9	0.00001
**Signs of severity**							
Polypnea	34	14.17%	124	12.41%	0	0	0.5
Cyanosis	5	2.08%	28	2.80%	0	0	0.0002
Oxygen saturation Spa02 < 95%	108	45.00%	416	41.64%	0	0	0.00001
Pneumonia	118	49.2	167	25.8	0	0	0.00001
Wheeze	52	21.67%	259	25,93%	0	0	0.0001
Tachycardia	35	14.58%	120	12.01%	0	0	0.000001
Hypotension	17	7.08%	45	4.50%	0	0	0.000001
Areas of mottled skin	9	3.75%	42	4.20%	0	0	0.000001
Discomfort	10	4.17%	39	3.90%	0	0	0.00002
Changes in mental status	12	5%	37	3.70%	0	0	0.00004
Oliguria	2	0.83%	5	0.50%	0	0	0.2
At least one of the above	177	73.8%	591	59.2%	0	0	0.00001

### Virological results

Virological samples were taken from 784/1,239 (63.3%) patients, as follows: CAP 177 (73.8%), SAS 447 (69.1%), and PSSI 160 (45.5%). Of these patients, 213 (27.2%) were positive for the influenza virus. The influenza positivity rate in each of the groups was as follows: CAP 52/177 (29.4%), SAS 115/447 (25.7%), and PSSI 46/160 (28.8%) (p = 0.6).

In the SAS group, the influenza positivity rates were according to the reason of presentation as follows: RF 76/263 (28.9%), HF 5/29 (17.2%), CF 15/68 (22.1%), and M 19/87 (21.8%) (p = 0.3). On the basis of the final diagnosis, the influenza virus positivity rates of the virological samples were as follows: asthma 60/231 (26%), AE-COPD 18/86 (20.9%), HIV 5/21 (23.8%), and acute cardiac failure 33/131 (25.2%).

The overall rate and the groups' positivity rates were not significantly different between the three-seasonal influenza epidemic periods (p = 0.5). The distribution of influenza strains among study groups is presented in Table 2.

**Table 2:** Influenza strains distribution among main study groups.

	Community-acquired pneumonia		Severe acute symptoms		Patients with underlying conditions for severe influenza		P
	n = 177		n = 447		n = 160		
	n	%	n	%	n	%	
Positive influenza	52	29.4	115	25.7	46	28.8	0.6
Influenza A	25	14.1	69	15.4	20	12.5	0.7
Influenza A H3N2	21	11.9	60	13.4	17	16.6	0.6
Influenza A H1N1	9	5.1	28	5.6	8	5	0,9
Influenza B	22	12.4	30	6.7	21	13.1	0.02

### Bacteriological results

Among the patients with virological samples, 92/784 (11.7%) had positive blood or respiratory bacteriological samples: Community acquired pneumonia: 32/177 (18%); Severe acute symptoms: 58/447 (13%); Patients with underlying conditions for severe influenza: 2/160 (1.3%). Streptococcus pneumonia (36/92 (39.1%) and Staphylococccus aureus 24/92 (26.1%) were the most common pathogens. Haemophilus influenza, Klebsiella pneumonia, Mycoplasma pneumonia, Pseudomonas aeruginosa and Streptococcus pyogenes explain the remaining isolates. Procalcitonin tests according to study groups were as follows (n (% of patients with procalcitonin >0.15μg/L), mean±SD (patients with procalcitonin >0.15μg/L): Community acquired pneumonia: 48/177 (27.1%), 0.1.9±2.2; Severe acute symptoms: 148/447 (33.1%), 2.2±4.1; Patients with underlying conditions for severe influenza: 24/160 (15%), 0.2±0.4. Both differences were significant between study groups (p = 0.000006 and p<0.0001).

### Severity criteria as a function of influenza infection

Comparisons of frequency of severity criteria with regard to Influenza virus virological results are presented in Table 3. There was no difference between patients with positive samples and those with negative samples.

**Table 3:** Frequency of severity criteria as a function of influenza results.

	Negative influenza		Positive influenza		P
	n = 571		n = 213		
	n	%	n	%	
Polypnea	82	14.4%	39	18.3%	0.2
Cyanosis	19	3.3%	6	2.8%	0.7
Oxygen saturation Spa02 < 95%	280	49%	107	50.2	0.8
Pneumonia symptoms and signs	149	26.1%	57	26.7%	0.3
Wheeze	162	28.4%	68	31.9%	0.3
Tachycardia	92	16.1%	26	12.2	0.2
Hypotension	35	6.1%	14	6.6%	0.8
Areas of mottled skin	27	4.7%	12	5.6%	0.6
Discomfort	20	3.5	9	4.2%	0.6
Changes in mental status	21	3.7%	13	6.1%	0.1
Oliguria	2	0.35%	3	1.4%	0.09
At least one of the above	401	70.2%	151	70.9	0,9
Positive bacteriological sample	31	5.4%	61	28.6%	0.000004

### Final disposition decision

Overall, 538/774 (71.1%) patients were admitted, 48 (6.2%) in ICU and 490 (63.3%) in medical ward. The ICU and medical ward admission rates as a function of study groups and Influenza results are presented in Table 4. There was no difference between the groups with regard to the virological results. Positive bacteriological sample and positive PCT value were not associated with final disposition decision (Table 4). In total, 11/784 (1.4%) patients died within the first 48 hours.

**Table 4:** Impact of influenza-positive virological samples on final disposition decision.

	Intensive care unit	Medical ward	Discharged	P
	n (%)	n (%)	n (%)	
All groups				0.5
Negative influenza	36 (6.3%)	361 (63.2%)	165 (28.9%)	
Positive influenza	12 (5.6%)	129 (60.6%)	71 (33.3%)	
Community-acquired pneumonia				0.9
Negative influenza	4 (3.2%)	91 (72.8%)	29 (23.2%)	
Positive influenza	2 (3.9%)	36 (69.2%)	14 (26.9%)	
Severe acute symptoms				0.5
Negative influenza	30 (9.3%)	213 (65.7%)	81 (25%)	
Positive influenza	9 (7.9%)	78 (68.4%)	27 (23.7%)	
Respiratory				0.8
Negative influenza	16 (8.8%)	121 (66.5%)	45 (24.7%)	
Positive influenza	8 (10.7%)	51 (68%)	16 (21.3%)	
Hemodynamic				0.5
Negative influenza	4 (17.4%)	14 (60.9%)	5 (21.7%)	
Positive influenza	1 (20%)	4 (80%)	0 (0%)	
Cardiac				0.2
Negative influenza	10 (19.2%)	31 (59.6%)	11 (21.2%)	
Positive influenza	0 (0%)	11 (73.3%)	4 (26.7%)	
Miscellaneous				0.8
Negative influenza	0 (0%)	47 (70.2%)	20 (29.9%)	
Positive influenza	0 (0%)	12 (63.2%)	7 (36.8%)	
Patients with underlying conditions for severe Influenza				0.1
Negative influenza	2 (1.8%)	57 (50%)	55 (48.3%)	
Positive influenza	1 (2.2%)	15 (32.6%)	30 (65.2%)	
Positive bacteriological sample				0.4
Negative influenza	22 (71%)	8 (25.6%)	1 (3.2%)	
Positive influenza	46 (75.4%)	14 (23%)	1 (1.6%)	

## Discussion

The results of our study indicate that the frequency of influenza-positive samples taken from symptomatic patients who presented at the ED was between 25.7% and 29.4%. Numerous clinical reasons for presentation at the ED were associated with influenza viruses, including acute decompensation of chronic cardiac and respiratory diseases, as well as acute episodes of community acquired pneumonia and respiratory and hemodynamic distress.

It has been reported that the frequency of the lung disease, the hospitalization rate and the mortality associated with Influenza-related pneumonia is increasing [20]. The frequency of lung damage is higher in patients with underlying cardiac or pulmonary diseases [21,22] and Influenza has been associated with increased mortality [23]. In the present study, 58% of the patients had at least one underlying condition for severe influenza [18], and 59% of patients had at least one severity criteria [19]. Respiratory distress was the main symptom, with up to 45% of patients exhibiting signs of clinically significant respiratory distress. It has previously been reported that 16% of cases of seasonal influenza occur in clinical pulmonary patients [24], 36% may develop acute pneumonia [24,25] and 20% may develop respiratory failure [25–28]. Our data support this finding, in that, among the proven cases of influenza virus, 49.2% had CAP, 25.8% had SAS with acute pneumonia, and up to 50% of patients had signs of respiratory distress. Neurological, hemodynamic, and cardiovascular complications have also previously been described in the context of severe infections that have been complicated by influenza [27,28]; in the present study, 12% of patients with influenza had hemodynamic disorders and 6% had neurological disorders. Some of the sepsis-like events that were observed in the group with hemodynamic failure could be related to frequent bacterial superinfection, which has been described in such patients [29].

Whereas only 5.6% of the patients with negative Influenza test had a bacterial coinfection, this figure reached 28.6% of patients with positive Influenza test. This feature indicates that Influenza may facilitate bacterial infection as previously resported [30]. Otherwise, bacterial coinfection was clearly more frequent in Community acquired pneumonia and Severe acute symptoms groups, both more frequently associated with severity criteria. However, coinfection was not associated with increased ICU and Medical ward admission rates. It has been reported that coinfection was frequently associated with severity criteria and ICU admission [31].

We found that the overall influenza positivity rate was 27.2%, and that, according to the reason for presentation in the ED (CAP 29.4%, SAS 25.7%, and PSSI 28.8%), the rates observed in the different groups were not significantly different. With regard to the final diagnostic categories (asthma (26%), EA-COPD (20.9%), HIV 5/21 (23.8%), and acute cardiac failure (25.2%) a high frequency was observed, but there was no difference between these categories. Our results indicate a higher frequency than that which was found in the few previous studies conducted in the ED, where, during the flu epidemic, the positivity rate of respiratory samples was low [32]. Our study shows that the positivity rates of respiratory specimens are very similar between patients with clinical symptoms suggestive of influenza than in patients with acute decompensation of chronic diseases. Most importantly, some of these clinical features are not usually recognized as being associated, or possibly associated, with the influenza virus.

It has previously been reported that the influenza virus has been isolated in 2.2% to 18% of people with CAP [11–13] and in 5% of people with severe acute respiratory infection admitted to ICU [33]. In the present study, the medical ward and ICU admission rates of people who had influenza-positive virus samples were 71.8% and 3.4%, respectively, while previously reported admission rates to MSW and ICU were 26% [12] and 15% [26,34], respectively. We found that the positivity of the influenza virus samples did not increase the frequency of the severity criteria or the rate of admissions to MSW or ICU, which corresponds to the results of previous studies [32,35]. An increase in ED attendance and hospitalizations due to decompensation of respiratory and cardiac pathologies during the winter period have previously been reported, but no direct link with epidemic influenza episodes was established [20,29]. Viruses can account for 30% of cardiac decompensations, but the influenza virus was isolated in only 3% of patients in an earlier study [14]. In the present study, up to 30% of episodes of acute cardiac failure, asthma, and AE-COPD were associated with the influenza virus. Our results indicate that influenza may account for 17%–29% of patients with severity criteria.

## Strengths and limitations

Ours was a monocentric study in an urban environment in a large city in Europe. Thus, our results cannot be generalized and require local assessments. It is accepted that clinical variables are insensitive and non-specific in predicting influenza [26,36–38], with worse results in adults and the elderly [34]. Therefore, among the non-tested patients, a number may have had undocumented influenza, which could alter the reported rates. The isolation of respiratory viruses is possible in asymptomatic individuals but Influenza detection is likely associated with the illness under evaluation [39]. Thus, positive samples can be considered clinically significant. Otherwise, the sample size was sufficient to evaluate the distribution of positive viral samples among the study groups, and the rates of inclusion and withdrawals appeared to be satisfactory.

## Conclusion

Our findings indicate that, during seasonal influenza epidemic episodes, 25–30% of emergency cases have positive influenza specimens, and this applies both to people experiencing symptoms that are suggestive of influenza and to people with acute respiratory infectious episodes, or episodes of respiratory, cardiac, or hemodynamic failure. High rates of medical ward and ICU hospitalizations amongst our entire population and the frequency of influenza virus among people whose clinical picture is not usually associated with influenza suggests that the risk of nosocomial influenza transmission must be considered as high during seasonal epidemic periods. As nosocomial influenza is currently recognized as an emerging concern [38], indications of isolation and treatment should be extended to these clinical situations during times of influenza epidemics. Our study thus opens up new prospects for research on indications of treatment and nosocomial transmission of influenza in the ED and hospital stay after through ED admission.
